# Correction: Study on static properties and mechanism of basalt fibre reinforced cement cured red sandstone soil

**DOI:** 10.1371/journal.pone.0340039

**Published:** 2026-01-02

**Authors:** Yao Long, Jun-Hua Chen, Jie-Jie Mu, Qi-Yun Wang

There is an error in affiliation 1 for author Yao Long. The correct affiliation 1 is: Hunan Provincial Engineering Research Center for Intelligent Operation and Maintenance of High-Speed Rail and Urban Rail Transit, Hunan Technical College of Railway High-speed, Hengyang 421002, China.

## References

[1] Long Y, Chen J-H, Mu J-J, Wang Q-Y. Study on static properties and mechanism of basalt fibre reinforced cement cured red sandstone soil. PLoS One. 2025;20(10):e0334316. doi: 10.1371/journal.pone.0334316 41160551 PMC12571302

