# Supplementary material for: Study on static properties and mechanism of basalt fibre reinforced cement cured red sandstone soil
Source: PLoS One. 2025 Oct 29;20(10):e0334316. doi: 10.1371/journal.pone.0334316 (PMC12571302; doi:10.1371/journal.pone.0334316)
Supplement: Attachment — Submitted filename: Response to Reviewers.docx [file pone.0334316.s004.docx]

Below are detailed comments and suggestions for improving the manuscript.

1. The study focuses on red sandstone from a specific location (Hengyang, Hunan). The paper should include a brief discussion about how applicable the results are to other red sandstone sources, which may vary in composition.

Re: agree with the changes. Additional clarification has been made in Conclusion (d).

2. All tests are performed under controlled lab conditions. Future work should include field tests or at least a discussion on how the results may translate to real-world roadbed performance.

Re: agree with the changes. It has been indicated in the Conclusion (d) how the improved technology can be applied to practical works.

3. Proofread carefully or use professional language editing to fix grammatical issues and polish technical writing.

Re: agree with the changes. The full text has been carefully proofread and grammatical issues have been corrected.

4. During disintegration test, you highlighted that C0BF0 specimen fully disintegrated after 11 minutes of water immersion, demonstrating very poor water stability. However, after cement modification, the water stability of the specimens improved significantly. The authors have not given deeper explanation on the mechanism which impacted specimen water stability when cement was added.

Re: agree with the changes. The mechanism of cement influence has been explained in further depth in Test results and analyses - Disintegration test.

5. Besides, the authors have concluded that, increasing the cement dosage also raises material brittleness and construction costs in red sand stone. Is the increase in brittleness specific to red sandstone soil, or is it consistent across different soil types? There’s an important point about cement increasing **brittleness** despite improving **strength** but it’s not deeply explored. Is there an optimal cement content where strength gains outweigh brittleness drawbacks?

Re: it is clear that the brittleness of different soils increases with the addition of cement, as has been illustrated in references 26-27. In addition, studies have been carried out on the causes of increased soil brittleness by cement as well as its manifestations, which are not the focus of this paper.

6. The results of Unconfined compressive strength (UCS) indicate with the increase in BF content from2‰ to 6‰, the UCS improved by 0.156 MPa, 0.378 MPa, and 0.765 MPa, representing increments of 5.18%, 12.55%, and 25.40%, respectively. However, when the BF content increased to 8‰, the UCS of the C4BF8 specimen was lower than that of C4BF6 specimen. There is no deeper explanation given regarding an increase in UCS and why the UCS decreased when 8% BF content was added. It would be beneficial to discuss how BF alters load transfer mechanisms, interfacial bonding, and structural integrity.

Re: a more in-depth explanation of the effect of BF content on ucs, and how BF alters the load transfer mechanism, interfacial bonding and structural integrity is in the Discussion section of the paper.

7. The study notes that at >6‰ BF, reinforcement declines due to uneven fiber distribution. It would be good to explore: Why this happens? is it mixing method, fiber length, or soil particle size?

Re: it's already been stated in the paper that it's excessive BF content.

8. The conclusion Lack of Quantitative Summary for All Results While a few results are backed with specific numbers (e.g., UCS increased by 25.40%, cohesion by 40.74%), many statements are qualitative**.** Example: “BF significantly enhanced the deformation and stability properties...” but **how much?** Consider revising the conclusion to include specific values (e.g., optimal content, measured strength increases) rather than general qualitative statements.

Re: agree with the change. Specific values have been added to the conclusions for clarification.

9. The conclusion ends by calling for more testing (dynamic loading, wet-dry cycles), which is good, but: It doesn’t provide practical guidelines for real-world use. For example, should engineers limit fiber content to 6? Should cement content not exceed 4% to avoid brittleness?

Re: agree with the change. Conclusion (d) has been given to give the mixing ratios that can be used in practice in specific projects.
